# Development of a Skewed Pipe Shear Connector for Precast Concrete Structures

**DOI:** 10.3390/ma10050531

**Published:** 2017-05-13

**Authors:** Sang-Hyo Kim, Jae-Gu Choi, Sejun Park, Hyunmin Lee, Won-Ho Heo

**Affiliations:** 1 School of Civil and Environmental Engineering, Yonsei University, Seoul 03722, Korea; sanghyo@yonsei.ac.kr (S.-H.K.); cjg1204@yonsei.ac.kr (J.-G.C.); sejooneee@yonsei.ac.kr (S.P.); 2 Railroad Infrastructure Research Team, Korea Railroad Research Institute, Gyeonggi-do 16105, Korea; hmin33@krri.re.kr

**Keywords:** shear connector, skewed pipe shear connector, push-out test, pull-out test, finite element analysis, precast concrete structure

## Abstract

Joint connection methods, such as shear key and loop bar, improve the structural performance of precast concrete structures; consequently, there is usually decreased workability or constructional efficiency. This paper proposes a high-efficiency skewed pipe shear connector. To resist shear and pull-out forces, the proposed connectors are placed diagonally between precast concrete segments and a cast-in-place concrete joint part on a girder. Design variables (such as the pipe diameter, length, and insertion angle) have been examined to investigate the connection performance of the proposed connector. The results of our testing indicate that the skewed pipe shear connectors have 50% higher ductility and a 15% higher ratio of maximum load to yield strength as compared to the corresponding parameters of the loop bar. Finite element analysis was used for validation. The resulting validation indicates that, compared to the loop bar, the skewed pipe shear connector has a higher ultimate shear and pull-out resistance. These results indicate that the skewed pipe shear connector demonstrates more idealized behavior than the loop bar in precast concrete structures.

## 1. Introduction

Precast concrete structures are widely used in buildings and bridges due to their economic and structural efficiency. In particular, multi-beam bridges fabricated from precast concrete box girders are one of the most commonly used bridge structures in the world. Such bridges constitute approximately one-sixth of the bridges built annually in the United States [1]. These types of structures are usually built using several adjacent precast or pre-stressed concrete segments, and can be constructed rapidly without requiring concurrent deck formation. Adjacent precast concrete boxes are typically connected by cast-in-place (CIP) joints placed in shear keys between each of the segments, and usually contain several high-strength tendons or threaded rods. In general, CIP joints serve two purposes in multi-beam box girder bridges. First, the joints prevent girder corrosion and block water leakage between the girders and consequently, the lower part of the bridge. Second, the joints prevent differential deflections of the girders and distribute vehicle loads equally across the entire bridge deck by achieving unified behavior of the girders [2]. Thus, the connecting system used in the CIP joints is extremely significant in the construction of multi-beam box structures. Ever since multi-beam bridges were introduced in the 1950s [3], researchers have studied and proposed improvements in the structural system as well as the connections between adjacent segments.

A shear key is a representative system that connects two precast segments, and various shapes and materials have been studied for this use. Box girders are generally connected using a shear key between each of the segments, with transverse tendons for lateral confinement [4]. Gulyas et al. [5] studied the use of a wide, full depth joint between precast units. This wider configuration accommodates panel tolerances more readily, and helps to ensure full grout-to-beam contact. Issa et al. [6] evaluated the performance of four different grout materials—polymer concrete for normal and hot conditions, and set grout concrete for normal and hot conditions—in precast concrete deck systems. Polymer concrete was found to be the best material for transverse joints in terms of strength, bonding, and failure mode. However, the authors recommended using set grout in transverse deck joints instead, simply because of its ease of use.

Recent investigations on multi-beam box girder bridges have revealed frequent longitudinal and reflective cracks in the grouted shear keys [7,8]. Loop bars that protrude from each side of the box girders and overlap with each other have been developed and employed to prevent this [9,10,11]. Longitudinal bars are placed through the loops, and CIP concrete is used to fill these joints. The use of loop bars improves the structure’s vertical shear resistance to loads and lateral pull-out resistance to bending moments. However, this complexity required to form this type of connecting system usually decreases the efficiency of construction. The required connections must be formed with couplers, and mutual interference problems are often encountered when placing the boxes. Hence, a new type of connecting system is needed for adjacent precast concrete box girders which will improve workability (time and effort of construction) as well as connection performance.

This paper proposes a novel connecting system called the skewed pipe shear connector for use in multi-beam box girder bridges. In this system, several steel pipes are placed at a skewed angle to ensure better connection performance with respect to the vertical shear resistances and lateral pull-out forces as well as improving workability. The load-carrying capacity of the skewed pipe shear connector is evaluated experimentally using a series of push-out and pull-out tests. In addition, finite element analysis assesses the effects of assorted variables affecting the connection performance of the proposed connector. A final conclusion section summarizes the results of this work.

## 2. Existing and Proposed Connecting System in Precast Multi-Beam Box Girders

**Figure 1:**
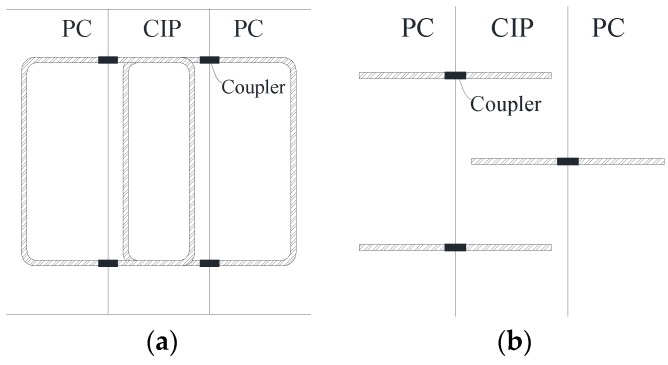
Installation overview of loop bars: (**a**) front view; (**b**) plan view.

Figure 1 illustrates the loop bar installation process. These are installed at the interface between adjacent box girders prior to girder placement. After the girders are placed and the formwork is dismantled, the loop bars from each segment are connected using couplers. This is inconvenient because each of these couplers must be installed by hand at every rebar joint. The couplers must also be protected before girders are place, and these protectors should be removed after finishing the formwork. In addition, all the couplers must be placed at the right locations for every joint longitudinally along the girder, a difficult task. Mutual interferences between adjacent girders may also occur when placing the girders. With these factors in mind, it can be said that the use of the loop bars in the CIP joints for multi-beam box girder bridges has some disadvantages in terms of ease of construction, while still showing structural advantages over the use of shear keys.

**Figure 2:**
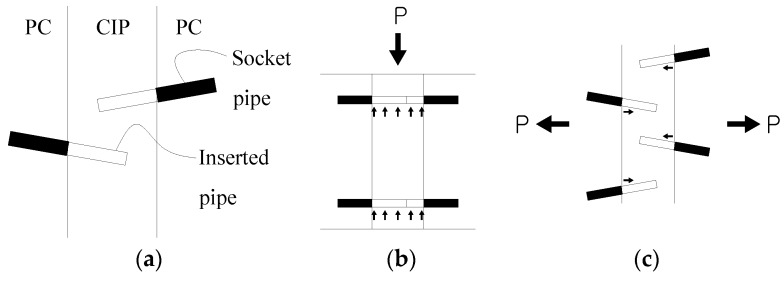
Installation overview and resistance mechanisms of skewed pipe shear connectors: (**a**) installation overview (plan view); (**b**) shear resistance (front view); (**c**) pull-out resistance (plan view).

**Figure 3:**
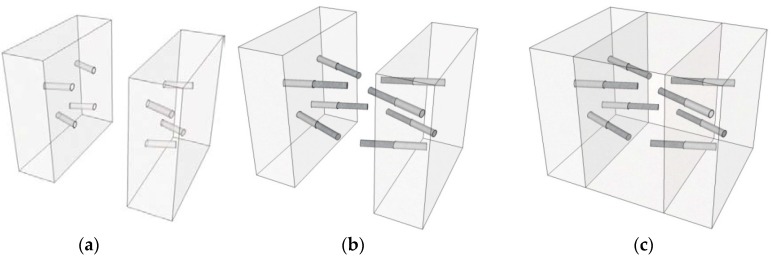
Installation process of skewed pipe shear connectors: (**a**) socket pipe installation, formwork, and concrete cast; (**b**) mold removal and pipe insertion; (**c**) pouring concrete (CIP).

In the proposed skewed pipe shear connector, steel pipes are embedded at a skewed angle in the interface between the adjacent precast concrete boxes. The novel technique proposed in this study addresses the problems of workability that occur when loop bars are used, and improves the structural shear and pull-out capacities. Figure 2 illustrates the schematic diagram and characteristics of the skewed pipe shear connector, consisting of a socket pipe and an inserted pipe. Figure 3 shows the installation process required for skewed pipe shear connectors. The socket pipes are installed in precast segments, while the inserted pipes are installed into the socket before pouring concrete is poured for the CIP joint section. The inserted and socket pipes are joined using epoxy mortar. This installation process reduces the time and effort of construction relative to the conventional loop bars placement method using couplers.

Skewed pipe shear connectors placed in the upper and lower parts of the interface between girders resist vertical shear forces, similar to conventional loop bars. Resistance to lateral pull-out forces is established by placing the pipe shear connecters diagonally in the transverse direction. In this study, diameters for the inserted and socket pipes were selected from conventional sizes of commercially available structural steel pipes with allowances of approximately 1.0 mm. The allowance was required to leave space for the epoxy mortar.

## 3. Experimental Tests for Assessing Shear and Pull-Out Resistances of the Skewed Pipe Shear Connector

### 3.1. Details of Experimental Tests

#### 3.1.1. Test Specimens

Various design variables that affect the connecting capacities of the skewed pipe shear connectors, including the pipe diameter, length, and insertion angle, were analyzed experimentally in this study. Table 1 summarizes the steel grades used in the skewed pipe shear connectors and loop bars, while Table 2 lists the dimension of the structural steel pipes. Two cases were considered. In the first case, the diameters of the shear connector and sockets were taken to be 42.7 mm and 48.6 mm, respectively, while the pipe thickness was 2.3 mm, and the allowance was 1.3 mm. In the second case, the diameters of the shear connector and sockets were set to 21.7 mm and 27.2 mm, respectively, while the pipe thickness was 2.3 mm, and the allowance was 0.9 mm.

Table 3 shows the variations between skewed pipe connectors used in the push- and pull-out tests that were performed to assess shear and pull-out resistances, respectively. The experimental construction for the skewed pipe shear connector used an insertion angle of 15°, and was composed of concrete with a compressive strength of 45 MPa. This concrete was chosen as it is typical for multi-beam box girder bridges that use loop bars. The diameter of insertion pipes was 42.7 mm (socket: 48.6 mm), the total length of inserted pipe was 400 mm (lengths of pipe inserted into the socket pipe and into the CIP are 200 mm each), and the thickness was 2.3 mm. For each variable condition, three specimens of the same specifications were manufactured to ensure the reliability of the test results.

**Figure 4:**
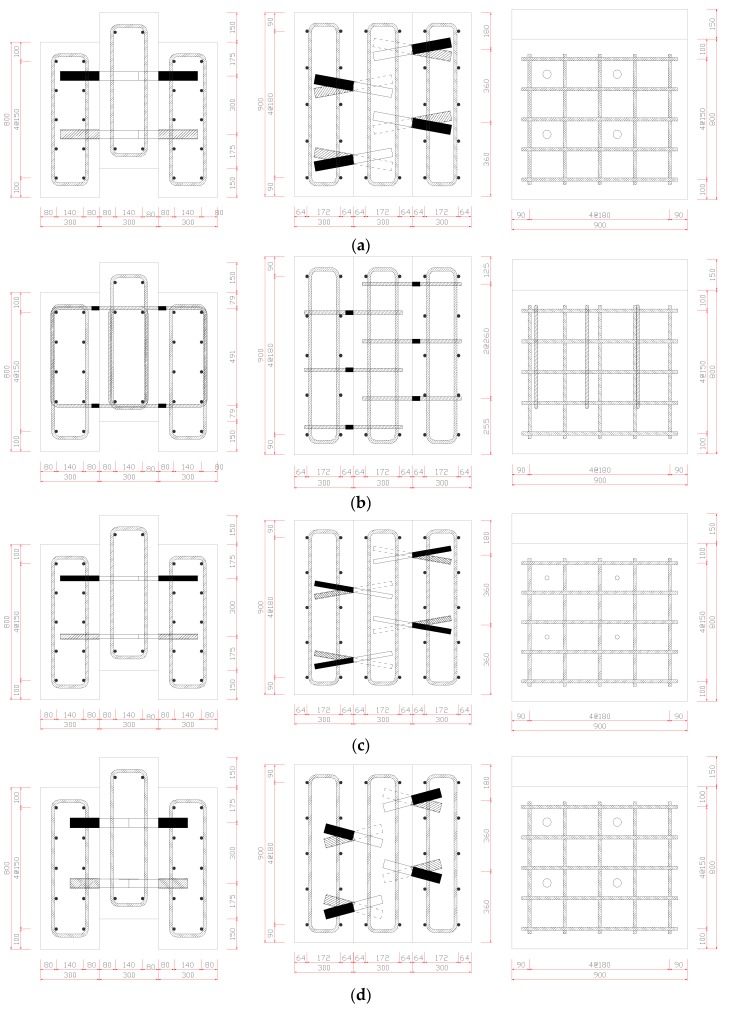
Layout of specimens for push-out tests (unit: mm): (**a**) skewed pipe shear connector (Reference); (**b**) loop bar; (**c**) skewed pipe shear connector (D = 21.7); (**d**) skewed pipe shear connector (L = 150).

**Figure 5:**
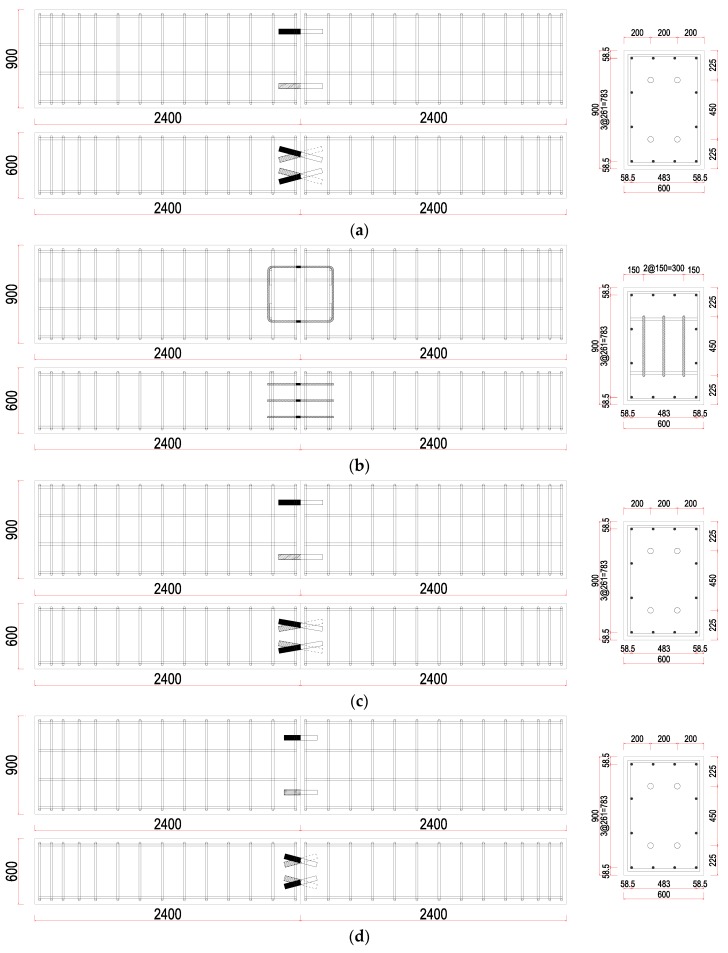
Layout of specimens for pull-out tests (unit: mm): (**a**) skewed pipe shear connector (Reference); (**b**) loop bar; (**c**) skewed pipe shear connector (A = 10); (**d**) skewed pipe shear connector (L = 150).

Experiments were carried with consideration of three parameters. First, the effect of the variation in the diameter of the skewed pipe shear connectors was evaluated by comparing pipes of diameter 42.7 mm (socket: 48.6 mm) and 21.7 mm (socket: 27.2 mm). Second, the effect of pipe length was evaluated by analyzing the results for pipes of lengths 200 mm and 150 mm. Finally, the effect of the insertion angle of skewed pipe shear connectors was evaluated by using insertion angles of 15° and 10°. The insertion angle of the pipe shear connector was not considered as a variable in push-out test because this does not significantly affect the shear resistance. In the push-out tests on the loop bar, the strength of concrete was applied in the same way as in the tests on the skewed pipe shear connector. This ensured that the changes in behavior could be observed under the same conditions. Figure 4 and Figure 5 show the details of each specimen.

#### 3.1.2. Push-Out Tests

**Figure 6:**
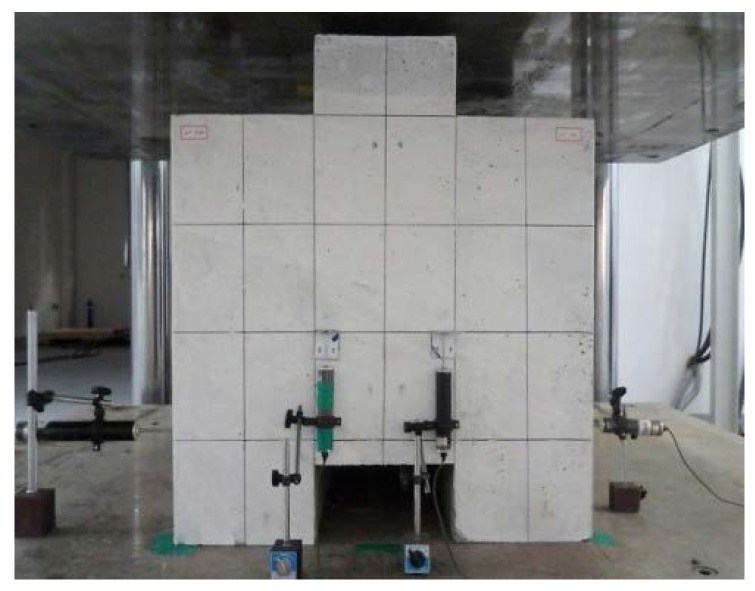
Push-out test set-up.

**Figure 7:**
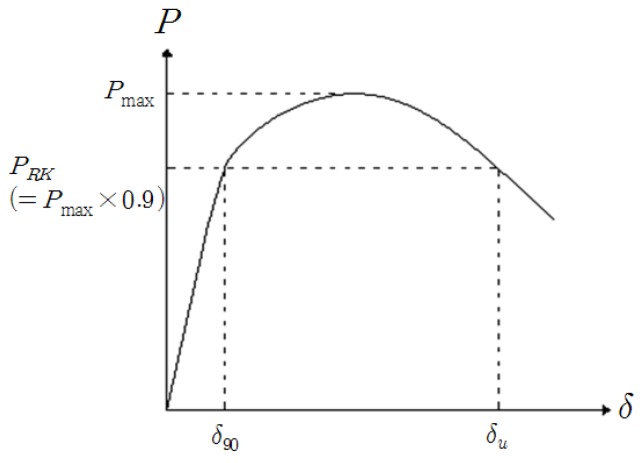
Evaluation method of test results.

In this study, the shear resistance was evaluated by conducting push-out tests, in which shear force is applied directly to the interface between concrete blocks. The shear resistance of the skewed pipe shear connector, alongside the change in the characteristics when variables were changed, was evaluated using the push-out tests proposed by Eurocode-4 [13]. A 5000 kN actuator was used for loading. To measure the relative slip between concrete blocks, a steel angle was attached 550 mm below the top of the concrete block and four 50 mm LVDTs (Linear Variable Differential Transformer) were installed. Loading was performed by displacement control in the vertical direction. The load speed was applied at a rate of 0.03 mm/s. The speed was controlled in accordance with Eurocode-4 to prevent the specimen from failing in under 15 min. Figure 6 shows the push-out test set-up. The push-out test results were evaluated based on the results of the relative displacement caused by loading. Figure 7 displays the criteria used for evaluating the results in this study [14]. Kim et al. [14] proposed the use of initial relative displacement (*δ*_90_) based on the characteristic load value (*P_RK_*), and compared the ratio of the slip capacity to initial relative displacement (*δ_u_*/*δ*_90_, for slip capacity *δ_u_*). As this ratio increases, so does the ductility of the shear connector relative to the initial stiffness. A safety factor was calculated using the ratio of the maximum load (*P*_max_) to the yield strength (*P_y_*).

#### 3.1.3. Pull-Out Tests

**Figure 8:**
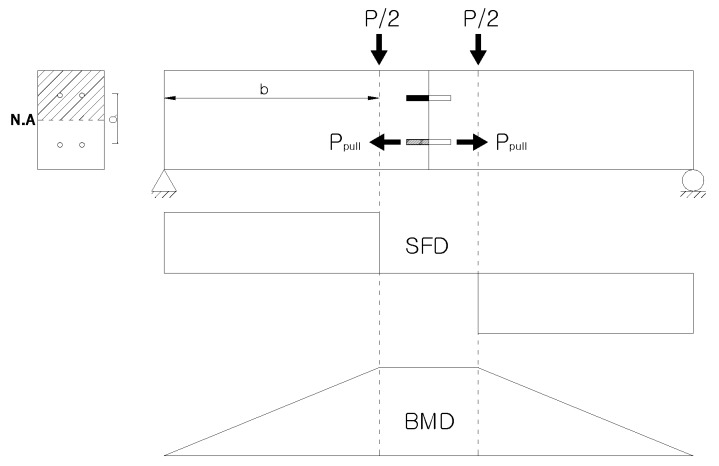
Overview of pull-out test.

**Figure 9:**
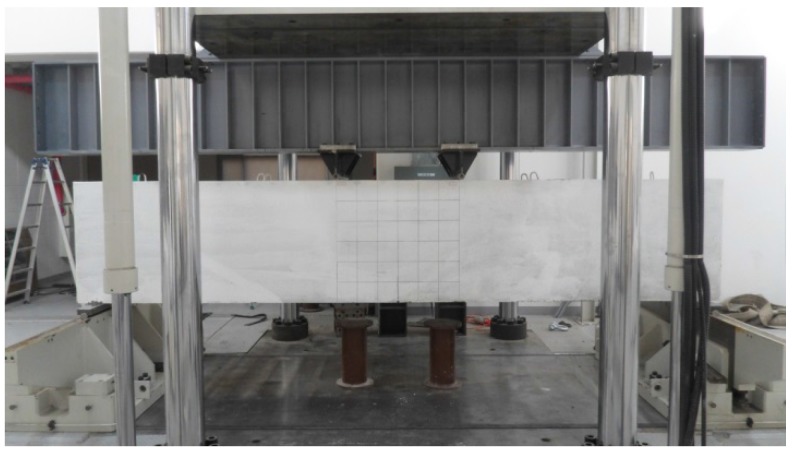
Pull-out test setup.

In this study, pure bending tests were carried out to evaluate the pull-out resistance performance of the skewed pipe shear connectors. As shown in Figure 8, a bending moment acts on the interface of a beam specimen that consists of two connected concrete blocks when vertical loads are applied on both sides of the interface. The bending moment is calculated as a function of the applied load, and the pull-out force acting on the connector is calculated from the bending moment (Equation (1)):

*P*_pull_ = *M*/*a* = *Pb*/2*a*, (1)

where *P*_pull_ (kN) is the pull-out force applied to the connector, *M* (kN m) is the moment acting on the interface of a specimen, *a* (m) is the distance from the center of the compressive zone to the center of the tensile zone of the connector, *P* (kN) is the vertical load acting on the specimen, and *b* (m) is the distance from the endpoint of the specimen to the point at which the vertical load acts. The pull-out force acting on the connector is the pull-out force on the connector located in the tensile zone, below the neutral axis of the cross section of the specimen. In this study, a connector was installed at the center of the compressive zone in each specimen. As a result, the moment arm length (*a*) was set equal to the distance between the centers of the connectors located in the compressive and tensile zones. Figure 9 shows the setup of the pull-out test.

### 3.2. Test Results

#### 3.2.1. Push-Out Test Results

Table 4 shows the load–relative displacement relationship obtained from push-out tests on three reference specimens using a skewed pipe shear connector. In each relationship, the presence of a ductile section after an initial linear section indicates ductile fracture behavior. The average maximum load of the three specimens was 775.5 kN. The difference between the specimens was less than 2%, proving the validity of the test results. The average ratio of slip capacity to the initial relative displacement, which is a measure of the ductility, was 2.8. The average ratio of maximum load to yield strength of the maximum load was 1.6. As shown in Figure 11, the three reference specimens were found to exhibit similar behaviors. The test results for the reference specimens and their respective parameter values were evaluated through comparison to the average values for the three specimens.

#### 3.2.2. Results of the Loop Bar Specimens in Push-Out Test

Table 5 show the load–relative displacement relationship for the three loop bar specimens. In contrast to the reference specimens, the loop bar specimens cracked after reaching the maximum load, exhibiting brittle fracture behavior with a rapid decrease in shear resistance. The average ultimate load of the three specimens was 918.8 kN. The difference between the specimens was less than 4%, validating the test results. The average ratio of slip capacity to initial relative displacement was 1.4, and the average ratio of maximum load to yield strength was 1.5. The average ultimate load is believed to have been large due to the effect of the 23 mm couplers.

**Figure 10:**
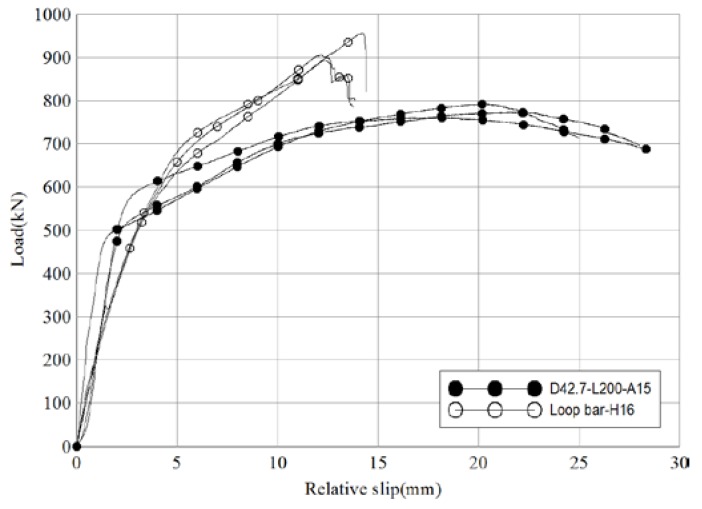
Comparison of load-relative slip relationships in pipe shear connectors and loop bars (push-out test).

Figure 10 and Table 6 compare the test results of the reference and loop bar specimens. The average maximum load of the reference specimens and loop bar specimens were 775.5 kN and 918.8 kN, respectively. The difference between these is insignificant, as it can be adjusted by changing the dimension of the loop bar or steel pipe used in a particular situation. In particular, the failure behavior characteristics of the shear connector were focused upon. Evaluation of sudden destruction and ductile behavior after the yield point is important for safety. As stated above, the average ratio of maximum load to yield strength was 1.6 for the reference specimens and 1.5 for loop bar specimens. The ratio of slip capacity to initial relative displacement was 2.8 for the reference specimens, 200% of the ratio shown by loop bar specimens (1.4). Therefore, it can be said that the skewed pipe shear connectors exhibited a similar ratio of maximum load to yield strength and better ductility in terms of ensuring the load-carrying capacity of the connections in which they were used when compared to the loop bars, and thus show superior performance.

#### 3.2.3. Effects of the Pipe Diameter (Inserted Pipe) in the Push-Out Test

Table 7 shows the load–relative displacement relationship for three specimens with a pipe diameter of 21.7 mm. Like the reference specimens above, these specimens exhibited characteristics of both rigid connectors and ductile connectors: a relatively short section of elastic strain behavior and a relatively long subsequent section of ductile behavior. The average maximum load of the three specimens was 537.9 kN. The difference between the specimens was less than 2%, proving the validity of the test results. The average ratio of slip capacity to the initial relative displacement was 2.0, and the average ratio of maximum load to yield strength was 1.5. The results were compared with the average values obtained from a reference specimen with a pipe diameter of 42.7 mm.

**Figure 11:**
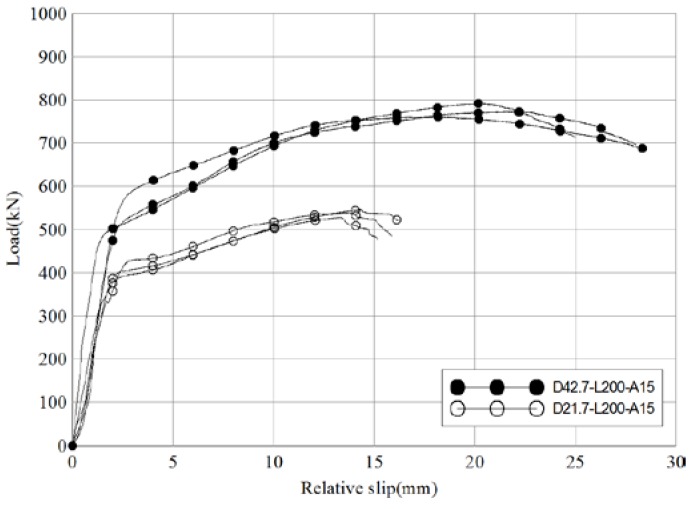
Comparison of load-relative slip relationships of different inserted pipe diameter specimens (push-out test).

Figure 11 and Table 8 compare the test results of D42.7-L200-A15 (reference diameter = 42.7 mm) and D21.7-L200-A15 (diameter = 21.7 mm). The cross-sectional area of the inserted pipe in D21.7-L200-A15 was 140.2 mm^2^, 48% of the reference specimen’s. Despite the change in the pipe diameter, the behavior patterns of the two specimens were found to be very similar. The average maximum load of D21.7-L200-A15 was 69% of the reference specimen’s (actual values 537.9 kN and 775.5 kN, respectively). The ratio of slip capacity to initial relative displacement was 2.8 for the reference specimen and 2.0 for D21.7-L200-A15 (70% of that of the reference specimen). Therefore, the diameter of the pipe is concluded to have a significant effect on skewed pipe shear connector performance with regards to shear resistance and ductility.

#### 3.2.4. Effects of Pipe Lengths (Inserted Pipe) in the Push-Out Test

Table 9 show the load-relative displacement relationship for three specimens with a total inserted pipe length of 300 mm (lengths of pipe inserted into the socket pipe and CIP are 150 mm each). The average ultimate load of the three specimens was 741.2 kN. The difference between the specimens was less than 2%, proving the validity of the test results. The average ratio of slip capacity to the initial relative displacement was 2.4, and the average ratio of maximum load to yield strength was 1.6. The results were compared with the average test results for the reference specimens with total inserted pipe lengths of 400 mm (lengths of pipe inserted into the socket pipe and into the CIP are 200 mm each).

**Figure 12:**
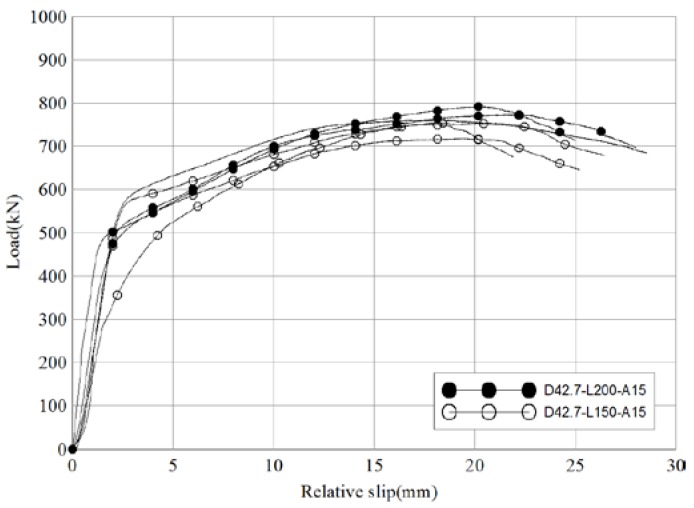
Comparison of load-relative slip relationships of different pipe length specimens (push-out test).

Figure 12 and Table 10 show the comparison of test results between D42.7-L200-A15 (lengths of pipes inserted into the socket pipe and into the CIP are 200 mm each) and D42.7-L150-A15 (lengths of the pipe inserted into the socket pipe and into the CIP are 150 mm each). Despite the differences in pipe length, the load-carrying capacities and behavioral patterns of the two sets of specimens are very similar. The average ultimate load of the reference specimens and D42.7-L150-A15 was 4% higher than that of the reference specimen (775.5 kN and 741.2 kN, respectively). The ratio of slip capacity to initial relative displacement was 2.8 for the reference specimens and 2.4 for D42.7-L150-A15 (86% of the reference specimens). These results show that the effect of pipe length on the shear resistance performance of skewed pipe shear connectors is insignificant, although ductility improves as the length of pipes increase.

### 3.3. Pull-Out Test Results

Table 11 shows the load-displacement results obtained from pull-out tests on three reference specimens using skewed pipe shear connectors. Ductile fracture behavior is evident throughout the ductile section that appears after the initial linear section. The three reference specimens had an average maximum load of 269.8 kN. The difference between the specimens was less than 4%, proving the validity of the test results. The average ratio of slip capacity to the initial relative displacement was 2.8. The pull-out resistance of the skewed pipe shear connector in the tensile zone, calculated from the maximum load and length of the moment arm, was 539.5 kN on an average. The average ratio of maximum load to yield strength of the maximum load to the yield load was 1.5. The test results for reference specimens were evaluated based on the average values for the three reference specimens.

#### 3.3.1. Results of the Loop Bar Specimens for Pull-Out Test

Table 12 show the load–displacement results for the three loop bar specimens. Unlike the skewed pipe shear connector specimens, the loop bar specimen exhibits brittle fracture behavior, losing load-carrying capacity rapidly and rupturing after reaching the maximum load. The three specimens exhibited an average maximum load of 321.4 kN. The average maximum load of the three specimens was 231.4 kN. The difference between the specimens was less than 6%, validating the test results. The average ratio of slip capacity to initial relative displacement was 1.9. The pull-out resistance of the loop bar located in the tensile zone, calculated from the ultimate load and moment arm length, averaged 642.8 kN. The average ratio of maximum load to yield strength was 1.3.

**Figure 13:**
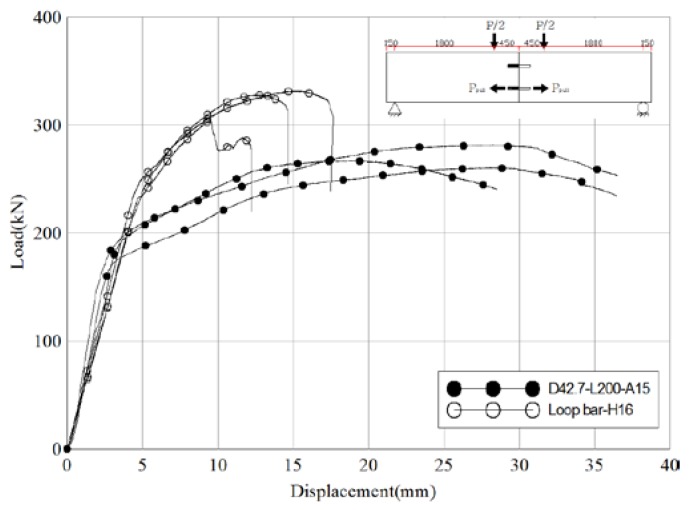
Comparison of load-displacements of different shear connector types (pull-out test).

Figure 13 and Table 13 compare the test results of the reference specimens and the loop bar specimens. The difference in the maximum load between the two is irrelevant, as it can be adjusted by changing on the dimension of the loop bar or steel pipe depending on the situation. Focus was placed on the failure behavior characteristics of the shear connector. The differences between sudden destruction and ductile behavior following the yield point are important for safety. The average ratio of maximum load to yield strength was 1.5 for the reference specimens and 1.3 for the loop bar specimens. The average ratio of slip capacity to initial relative displacement was 2.8 for the reference specimens and 1.9 for the loop bar specimens (68% of that of the reference specimens). These results are indicative of brittle fracture behavior, suggesting rebar will rupture in the case of the loop bar. Therefore, it can be conjectured that skewed pipe shear connectors display ductile failure behavior at a similar level of the ratio of maximum load to yield strength when loop bars undergo brittle fracture behavior. This shows that skewed pipe shear connectors can ensure higher safety factor after yield strength in comparison to loop bars.

#### 3.3.2. Effects of the Pipe Insertion Angles in Pull-Out Test

Table 14 shows the load–displacement results for three specimens with a pipe insertion angle of 10°. Ductile fracture behavior was exhibited, as in the case of the reference specimens. The average ultimate load of the three specimens was 256.1 kN. The difference between the specimens was less than 2%, proving the validity of the test results. The average ratio of slip capacity to initial relative displacement was 2.8. The pull-out resistance of the skewed pipe shear connector located at the tensile zone, calculated from the ultimate load and moment arm length, was 512.2 kN on an average. The average ratio of maximum load to yield strength was 1.6. The results were compared with the average results for the reference specimens with a pipe insertion angle of 15°.

**Figure 14:**
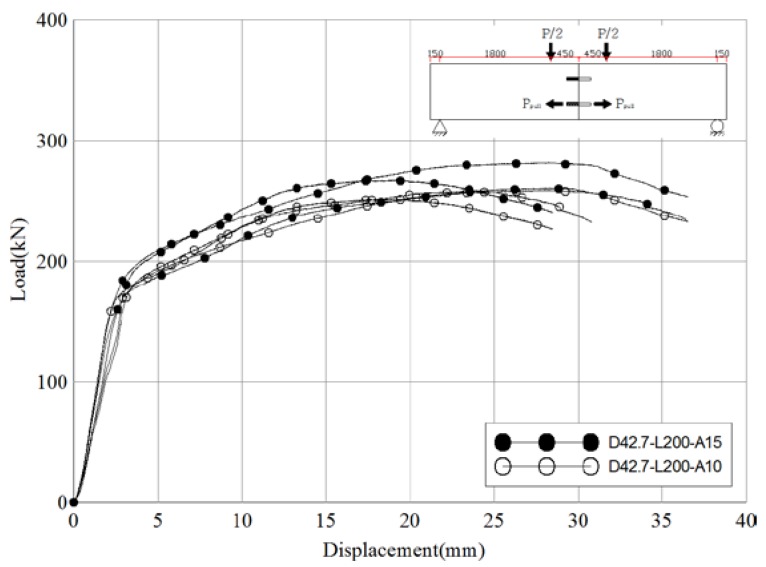
Comparison of load-displacement of different pipe insertion angle specimens (pull-out test).

Figure 14 and Table 15 compare the test results of D42.7-L200-A15 (reference angle = 15°) and D42.7-L200-A10 (angel = 10°). Even with the change in the insertion angle of the pipe, the behavioral patterns and results for the two sets of specimens were closely related. The ratio of slip capacity to the initial relative displacement was 2.8 for the reference specimens and 2.8 for D42.7-L200-A10. The average pull-out resistance of the skewed pipe shear connectors was 539.5 kN for the reference specimens and 512.2 kN for D42.7-L200-A10 (a difference of approximately 5%). These results indicate that a 5° change in the pipe insertion angle does not have a significant effect on the pull-out performance of skewed pipe shear connectors. This can be considered advantageous, as construction errors in terms of the insertion angle of the pipes will not have a significant effect on performance or safety.

#### 3.3.3. Effects of the Pipe Lengths (Inserted Pipe) in the Pull-Out Test

Table 16 show the load–displacement results for three specimens with total inserted pipe lengths of 300 mm (lengths of pipes inserted into the socket pipe and into the CIP are 150 mm each). The average ultimate load of the three specimens was 167.0 kN. The difference between the specimens was less than 6%, validating the test results. The average ratio of slip capacity to initial relative displacement was 2.6. The average pull-out resistance of the skewed pipe shear connector located in the tensile zone, calculated from an ultimate load and moment arm length, was 334.1 kN. The results were compared with the average results for reference specimens with a total inserted pipe length of 400 mm (lengths of pipes inserted into the socket pipe and into the CIP are 200 mm each).

**Figure 15:**
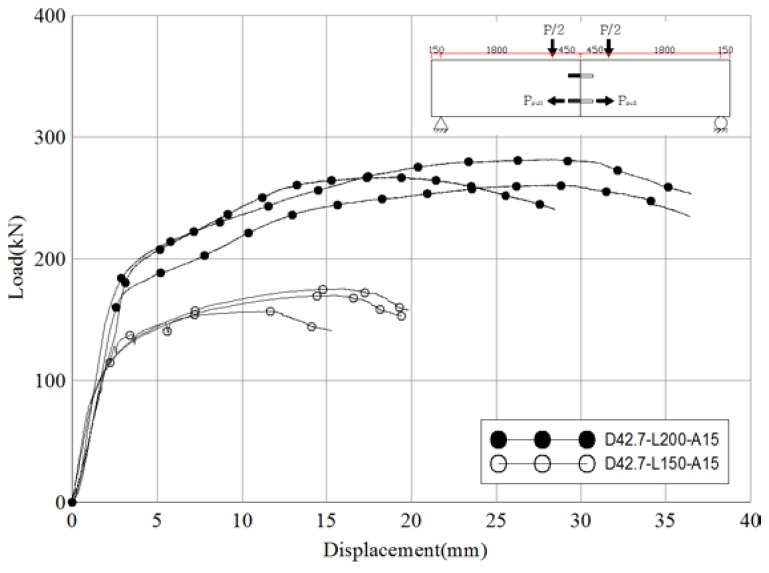
Comparison of load-displacement of different pipe length specimens (pull-out test).

Figure 15 and Table 17 compare the test results for D42.7-L200-A15 (reference lengths of pipes inserted into the socket pipe and into the CIP are 200 mm each) and D42.7-L150-A15 (lengths of pipes inserted into the socket pipe and into the CIP are 150 mm each). Large differences were evident between the results for the two sets of specimens. The ratio of slip capacity to the initial relative displacement was 2.8 for the reference specimens and 3.1 for D42.7-L150-A15, indicating similar ductility. However, the average pull-out resistance of the skewed pipe shear connectors was 539.5 kN for the reference specimens, whereas it was 334.1 kN for D42.7-L150-A15 (a 38% difference). These results indicate that the pipe length has a large effect on the pull-out resistance performance of skewed pipe shear connectors.

### 3.4. Failure of the Specimens

#### 3.4.1. Shear Failure

**Figure 16:**
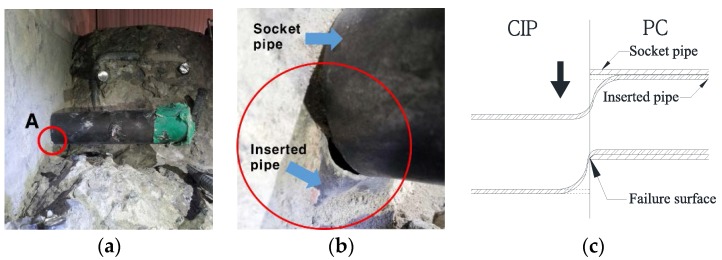
Shear failure of a skewed pipe shear connector: (**a**) shape of failure; (**b**) close-up: part A; (**c**) overview of shear failure.

**Figure 17:**
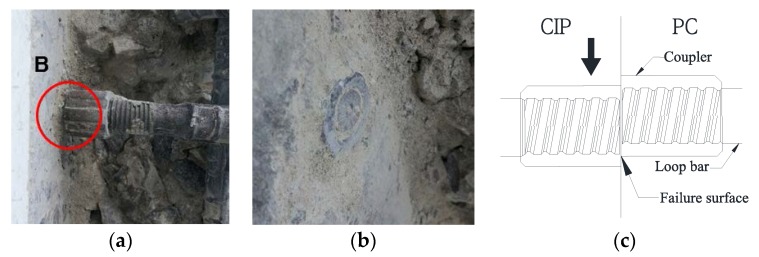
Shear failure of a loop bar: (**a**) shape of failure; (**b**) close-up: part B; (**c**) overview of shear failure.

In this section, failure patterns of separate specimens using a fabricated skewed pipe shear connector and loop bar were examined. In both the specimens, no cracks were observed in the external side of the concrete blocks. This seems to be a result of the loss in the shear resistance capacity of both connectors prior to concrete failure. After the push-out test, the concrete blocks of the specimen were cut to check the shape connector failures, shown in Figure 16 and Figure 17. The skewed pipe shear connector exhibited bending deflection along the line of interface between concrete blocks. This indicates that the shearing force on the interface caused the deformation, leading to the skewed pipe shear connector’s ductile failure behavior. Unlike the skewed pipe shear connector, the loop bar had no bending deflection. This indicates that when the loop bar and its coupler failed, the connector rapidly lost shearing resistance performance at the maximum load. These results imply that the material properties of connectors significantly influence their shearing resistance performance.

#### 3.4.2. Tensile (Pull-Out) Failure

**Figure 18:**
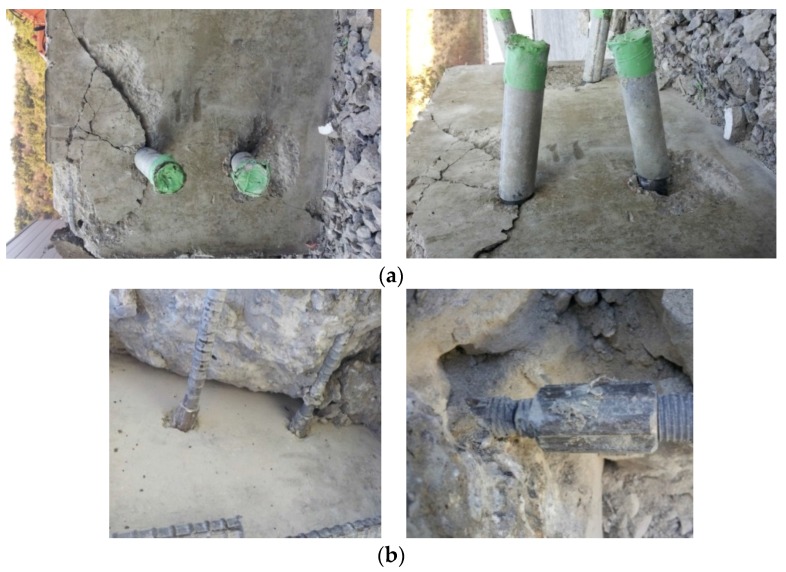
Crack shape after pull-out test: (**a**) skewed pipe shear connector specimen; (**b**) loop bar specimen.

There were no cracks in the tensile and compressive zones on the external sides of the skewed pipe shear connector or loop bar specimens. After the pull-out test, the concrete blocks of the specimen were cut to check the failure and deformation shape of the concrete blocks and connectors. Figure 18a shows the shape of failure of the skewed pipe shear connector. As shown in the figure, a failure surface was created at approximately 45° to the direction in which the pipe-typed shear connector was inserted. In addition, a slight deformation was observed in the direction of the pull-out force applied to the concrete blocks. This shows that the pull-out resistance of the skewed pipe shear connector was affected by variations in the pipe insertion angle as well as the concrete strength. Figure 18b shows the shape of failure of the loop bar specimen. Unlike the skewed pipe shear connector, this specimen showed no failure surface. However, failure of the loop bar occurred around its coupler. This implies that the pull-out resistance of the loop bar was significantly influenced by the tensile resistance of loop bar, rather than the resistance of the concrete blocks.

## 4. Finite Element Analysis for Skewed Pipe Shear Connector

### 4.1. Analysis Method

**Figure 19:**
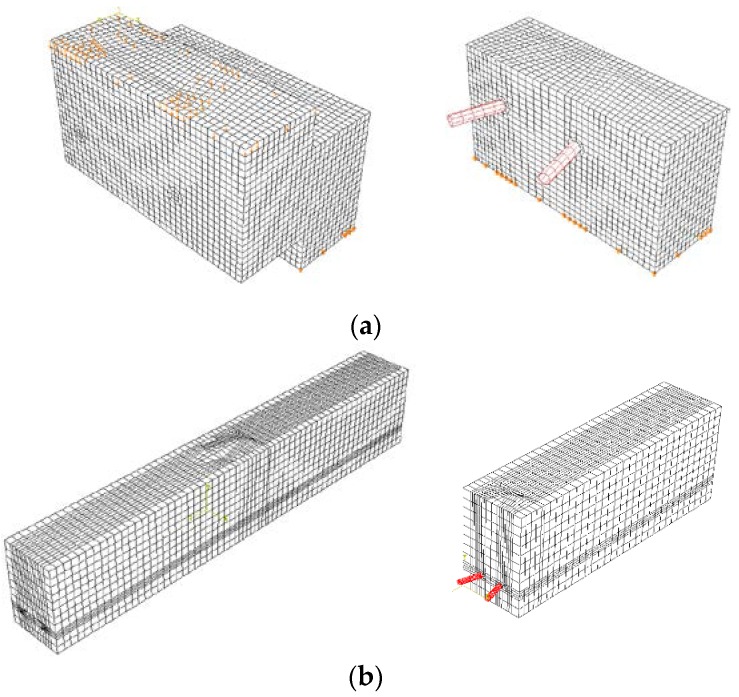
Finite element mesh: (**a**) model of push-out test specimen; (**b**) model of pull-out test specimen.

This section describes the numerical simulations conducted to evaluate the shear resistance and pull-out resistance for push-out specimens with both the skewed pipe shear connector and loop bar. Taking advantage of symmetry to reduce the size of the problem, only one-half of the push-out specimen was modeled. At least two skewed pipe were required to demonstrate the performance of the shear resistance and pull-out resistance. Thus, the numerical simulations that evaluate the shear and pull-out performance were composed of two skewed pipe shear connectors. The results of the analysis were compared with the results of the experiment using multiplication. The finite element mesh of these specimens is presented in Figure 19.

**Figure 20:**
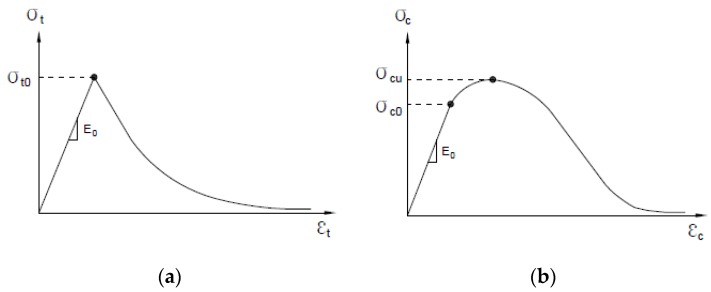
Stress–strain relationship of the concrete damaged plasticity model: (**a**) uniaxial tension model; (**b**) uniaxial compression model.

**Figure 21:**
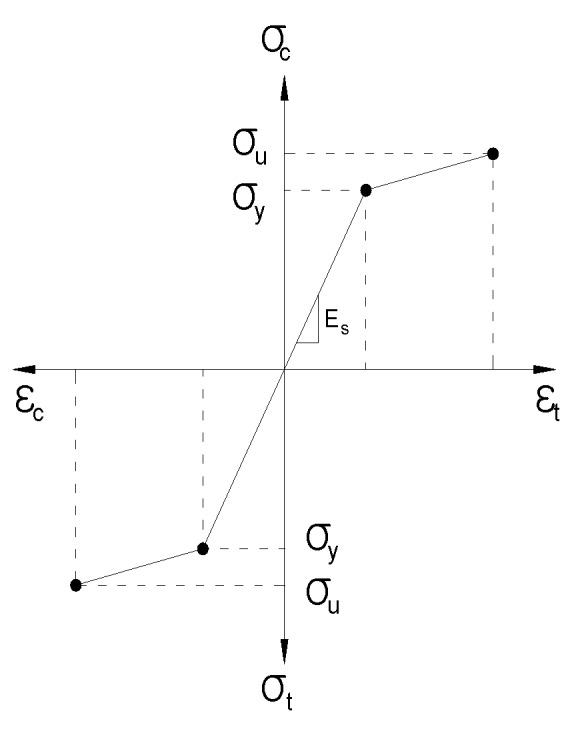
Stress–strain relationship of classical metal plastic model.

Concrete and steel skewed pipe shear connectors were modeled using solid and shell elements, respectively. Shell elements were analyzed by changing the attributes for each thickness of pipe. They were applied to offset the top side to prevent combination with the concrete elements. A concrete damaged plasticity model, suitable for modeling a material that exhibits different yield strength in tension and compression, was used to simulate the concrete material. The suitability of this model is demonstrated in Figure 20. A classical metal-plastic model was applied to the steel that exhibited a bi-linear stress–strain relationship with respect to compression and tension, as shown in Figure 21. In this study, the basic behavior of the skewed pipe shear connector specimen was assumed to exhibit linear behavior prior to yield stress. Following yield stress, it was assumed to exhibit perfectly plastic behavior.

To consider only resistance due to the insertion angle of the pipe, the tangential behavior of the concrete contact surface was assumed to be frictionless. In addition, the contact algorithm provided by ABAQUS (6.12, Dassault Systèmes, Tokyo, Japans) and empirical coefficient of friction were used to prevent penetrations between the steel and concrete materials. This allowed for an efficient transfer of load in a direction normal to the boundary interface. Loading was conducted by controlling the displacement in both the push-out and pull-out tests, setting these to 40 mm and 30 mm, respectively. The loads were calculated using the reaction force of the loading point, and analysis results were derived from its displacement value.

### 4.2. Validation of Numerical Model

#### 4.2.1. Push-Out Simulations

**Figure 22:**
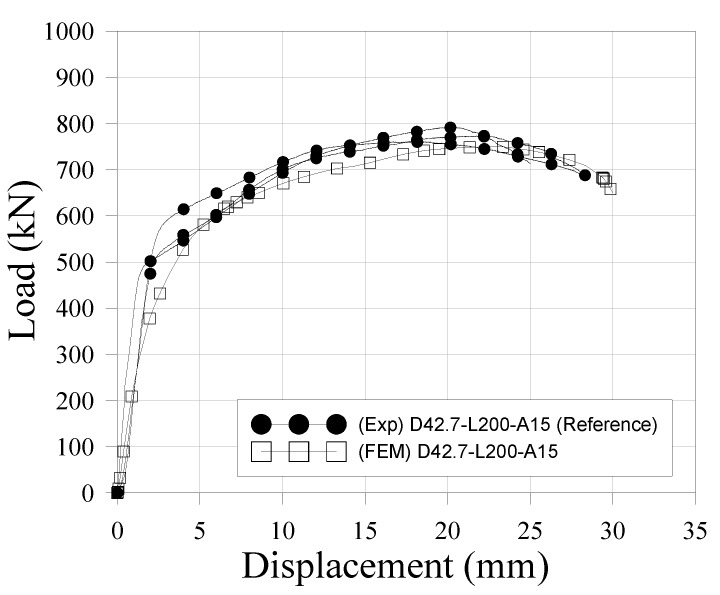
Load-relative slip characteristics of push-out test.

The numerical model was validated by comparing its performance with the push-out test results. The load-relative slip characteristics obtained from finite element analysis are shown in Figure 22, where the push-out test results for reference specimens (D42.7-L200-A15) are also plotted. Table 18 summarizes the results from the experimental tests and finite elements method (FEM). The ultimate loads show a similar result and represent a conservative trend compared to the experimental results. Similarly, the initial stiffness of the FEM, which can be described as the initial slope of the curve, is located slightly below the experimental curve. When considering the aspect of safety design, the numerical model is in good agreement with the test results. These results are consistent with and validate the proposed numerical model.

#### 4.2.2. Parametric Study with Numerical Simulations (Push-Out)

**Figure 23:**
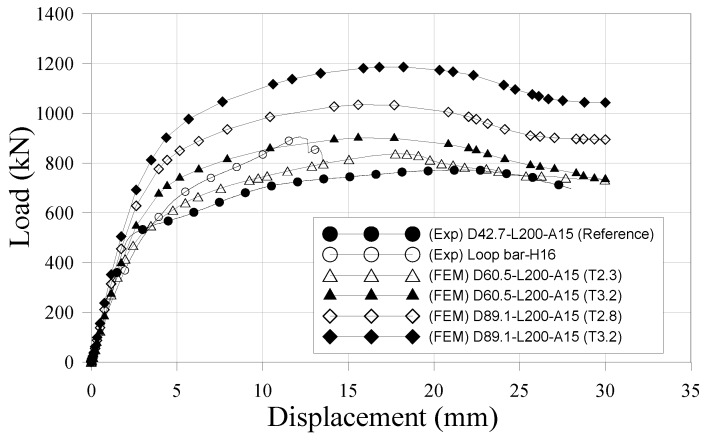
Comparison of experimental and numerical results (push-out test).

Numerical simulations were used to evaluate the effects of more assorted variables, specifically, pipe diameters of 60.5 mm and 89.1 mm, and pipe thickness of 2.3 mm and 3.2 mm. The numerical models were compared with the experimental results of reference specimen and loop bar specimen. Figure 23 shows the load-slip curves. Table 19 summarizes all the results from the experimental tests and numerical model. In Figure 23, the loop bar specimen has a maximum load higher than that of the reference specimen. However, the ultimate loads of the skewed pipe shear connector specimens gradually increase with as pipe diameter and thickness increase. D89.1-L200-A15 (T3.2) had a maximum load of 1186.5 kN at a relative displacement of 17.8 mm, and the loop bar had ultimate maximum load of 918.8 kN at a relative displacement of 12.9 mm (77% of that of D89.1-L200-A15 (T3.2)). The ratio of the slip capacity to the initial relative displacement was 3.2 for D89.1-L200-A15 (T3.2) and 1.4 for the loop bar (44% of that of D89.1-L200-A15 (T3.2)). Therefore, the results can be said to show skewed pipe shear connectors are superior to loop bars in terms of ensuring the load-carrying capacity of the connections in which they are used.

#### 4.2.3. Pull-Out Simulations

**Figure 24:**
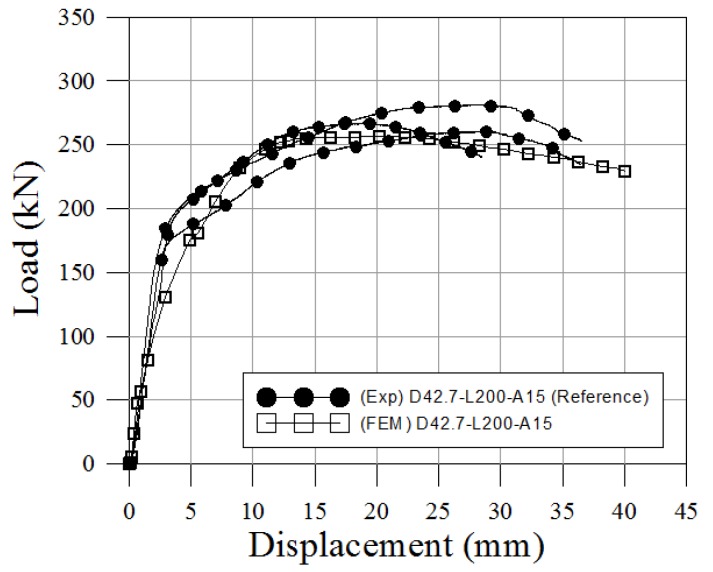
Load-displacements of the pull-out test.

The numerical model is validated by comparing its findings with the pull-out test results. The load-relative slip characteristics obtained from finite element analysis are shown in Figure 24, in which the pull-out test results for reference specimens (D42.7-L200-A15) are also plotted. Table 20 summarizes the results from the experimental tests and the numerical model. The numerical model had an ultimate load of 512.8 kN (95% of that of the reference specimen). The ratio of slip capacity to the initial relative displacement was 4.6 for the numerical model (164% of that of the reference specimen). This can be attributed to the separation of epoxy mortar used for pipe adhesive from the socket when pull-out force acts. The reason seems to be that oil was not completely removed from the inside of the pipe at the time of the specimen production. However, the overall shape and maximum pull-out strength of the load-displacement were interpreted at an appropriate level. Therefore, the analysis results were valid.

#### 4.2.4. Parametric Study with Numerical Simulations (Pull-Out)

**Figure 25:**
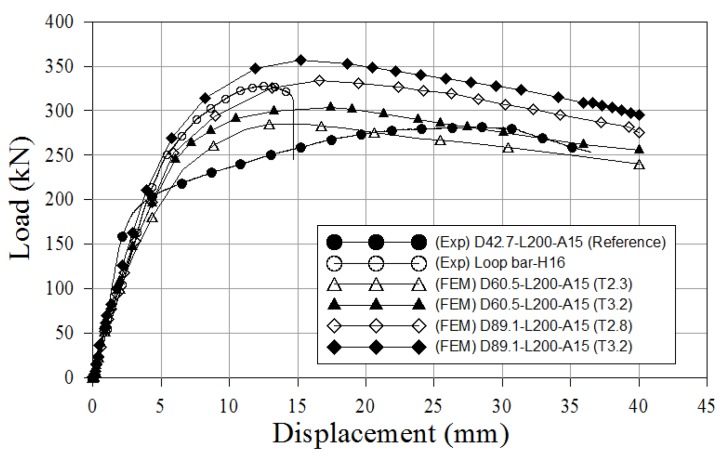
Comparison of experimental and numerical results (pull-out test).

Numerical simulations were used to evaluate the effects of more assorted variables, specifically, pipe diameters of 60.5 mm and 89.1 mm, and pipe thickness of 2.3 mm and 3.2 mm. The numerical models were compared to the experimental results of reference specimen and loop bar specimen. Figure 25 shows the load-displacement curves. Table 21 summarizes all the results from the experimental tests and the numerical model. In Figure 25, the loop bar specimen has an ultimate load higher than that of the reference specimen. However, the ultimate loads of the skewed pipe shear connector specimens gradually increase as the pipe diameter and thickness increase. D89.1-L200-A15 (T3.2) had an ultimate load of 713.2 kN, whereas the loop bar had an ultimate load of 642.8 kN (90% of that of D89.1-L200-A15 (T3.2)). The ratio of slip capacity to initial relative displacement was 3.5 for D89.1-L200-A15 (T3.2) and 1.9 for the loop bar (54% of that of D89.1-L200-A15 (T3.2)). Therefore, the skewed pipe shear connectors seem to be superior to the loop bars in terms of ensuring the load-carrying capacity of the connections in which they were used.

## 5. Conclusions

This study proposed a skewed pipe shear connector that can be applied to various types of concrete structures which improved workability and ductility behavior over the currently used construction methods. Analyses of the characteristics of this connector were performed by the use of push-out tests, pull-out tests, and finite element analysis. Shear connector specimens were fabricated according to Eurocode-4 requirements and push-out tests were conducted to evaluate the variation in shear resistance according to the pipe diameter and length. Pipe diameters from 21.7 mm to 42.7 mm and lengths from 150 mm to 200 mm were considered as variables for evaluating the effect of the design variables on the skewed pipe shear connector. Pull-out tests were conducted on the skewed pipe shear connector to evaluate the variation in the pull-out resistance according to the pipe insertion angle and length. Pipe insertion angles from 10° to 15° and lengths from 150 mm to 200 mm were considered as variables for evaluating the effect of design variables on the skewed pipe shear connector. In addition, prepared specimens were used to compare the behavior of the skewed pipe shear connector with that of loop bar. Moreover, finite element analysis was conducted to evaluate the shear resistance and pull-out resistance performance according to the variation in pipe diameter and thickness. The conclusions obtained in this study are summarized below.

The results of the push-out tests and pull-out tests indicated that the skewed pipe shear connector has better ductility than the loop bar. The skewed pipe shear connector showed 50% higher ductility. Furthermore, the ratio of maximum load to yield strength of the skewed pipe shear connector was 15% larger than that of the loop bar. Therefore, the skewed pipe shear connector demonstrated more ideal behavior than the loop bar in terms of ductile behavior.

The evaluation of behavioral changes of the skewed pipe shear connector demonstrated that when the pipe diameter increased from 21.7 mm to 42.7 mm, the shear resistance of the skewed pipe shear connector increased by 44%. Thus, it was shown that the pipe diameter significantly affects the shear resistance of the skewed pipe shear connector. When the pipe length increased, the pull-out resistance and ductility of the skewed pipe shear connector increased, showing that increasing the pipe length improved the pull-out performance and ductility.

The results of the finite element analysis indicated that shear resistance and pull-out resistance gradually increase with increases in the pipe diameter and thickness. In addition, the skewed pipe shear connector exhibited a maximum shear resistance and pull-out resistance higher than that of the loop bar when the pipe diameter and thickness increased.

From the experimental and numerical evaluations undertaken in this study, it was demonstrated that the skewed pipe shear connector has better ductility and higher shear resistance than the traditionally used loop bar. Moreover, the skewed pipe shear connector has improved workability and pull-out resistance relative to the loop bar. However, those effects might differ from structure to structure, since the structural performance of the skewed pipe shear connectors depends strongly upon the details of a structural system, including pre-stressing effects, material properties, and dimensions of the pipes. Additional studies on those factors are therefore in order. Methods for improving frictional resistance for the connectors will also be considered as a practical use of the skewed pipe shear connectors.

## Figures and Tables

**Table 1:** Material properties of the connectors [12].

Connectors	Steel Grade	Yield Strength (MPa)	Tensile Strength (MPa)
Loop bar	SD 400	400	560
Skewed pipe shear connector	STK 400	235	400

**Table 2:** Dimensions of the structural steel pipe.

Pipe Type	External Diameter (mm)	Thickness (mm)	Internal Diameter (mm)	Cross-Section Area (mm^2^)
Inserted	21.7	2.3	17.1	140.2
Socket	27.2	2.3	22.6	179.9
Inserted	42.7	2.3	38.1	291.9
Socket	48.6	2.3	44.0	334.5

**Table 3:** Variation in the skewed pipe shear connectors for push- and pull-out test specimens.

Specimens	Socket Length (mm)	Inserted Pipe Length (mm)		Insertion Angle (°)	No. of Connectors ** Installed	Concrete Comp. Strength (MPa)	Quantity of Specimens	
		Into the Socket	Into the CIP				Push-Out	Pull-Out
D42.7-L200-A15 * (Reference)	200	200	200	15	8/4	45	3	3
Loop bar-H16	N.A. *** (dia. of loop bar = 16 mm)				12/6	45	3	3
D21.7-L200-A15	200	200	200	15	8/N.A.	45	3	N.A.
D42.7-L150-A15	150	150	150	15	8/4	45	3	3
D42.7-L200-A10	200	200	200	10	N.A./10	45	N.A.	3

* D: Diameter (mm), L: Total length of inserted pipe (mm), and A: Insertion angle (°); ** Number of shear connectors for push-out specimen/pull-out specimen; *** Not Applicable.

**Table 4:** Results of push-out test (D42.7-L200-A15).

Specimens	No.	*P*_max_ (kN)	*P_y_* (kN)	*P_RK_* (kN)	*δ*_90_ (mm)	*δ_u_* (mm)	*δ_u_*/*δ*_90_	*P*_max_/*P_y_*
D42.7-L200-A15 (Reference)	1	773.3	488.2	696.0	9.8	28.0	2.9	1.6
	2	792.0	405.9	712.8	11.0	25.0	2.3	2.0
	3	761.2	551.9	685.1	8.2	28.5	3.5	1.4
	Avg.	775.5	482.0	698.0	9.6	27.2	2.8	1.6

**Table 5:** Results of push-out test (loop bar).

Specimens	No.	*P*_max_ (kN)	*P_y_* (kN)	*P_RK_* (kN)	*δ*_90_ (mm)	*δ_u_* (mm)	*δ_u_/δ*_90_	*P*_max_/*P_y_*
Loop bar-H16	1	906.0	579.2	815.4	9.4	13.5	1.4	1.6
	2	894.3	552.2	804.8	9.8	14.6	1.5	1.6
	3	956.1	648.8	860.5	11.3	14.4	1.3	1.5
	Avg.	918.8	593.4	826.9	10.2	14.2	1.4	1.5

**Table 6:** Comparison of the characteristic values of different types of shear connector specimens (push-out test).

Specimens	*P*_max_ (kN)	*P_y_* (kN)	*P_RK_* (kN)	*δ*_90_ (mm)	*δ_u_* (mm)	*δ_u_*/*δ*_90_	*P*_max_/*P_y_*
(A) Loop bar-H16	918.8	593.4	826.9	10.2	14.2	1.4	1.5
(B) D42.7-L200-A15 (Reference)	775.5	482.0	698.0	9.5	27.2	2.8	1.6
Ratio (A/B)	1.19	1.23	-	-	-	0.49	0.94

**Table 7:** Results of push-out test (D21.7-L200-A15).

Specimens	No.	*P*_max_ (kN)	*P_y_* (kN)	*P_RK_* (kN)	*δ*_90_ (mm)	*δ_u_* (mm)	*δ_u_*/*δ*_90_	*P*_max_/*P_y_*
D21.7-L200-A15	1	539.5	394.3	485.6	7.3	15.9	2.2	1.4
	2	528.3	338.0	475.5	8.1	15.2	1.9	1.6
	3	546.0	376.8	491.4	9.1	17.3	1.9	1.4
	Avg.	537.9	369.7	484.1	8.2	16.1	2.0	1.5

**Table 8:** Comparison of characteristic values of different inserted pipe diameter specimens (push-out test).

Specimens	*P*_max_ (kN)	*P_y_* (kN)	*P_RK_* (kN)	*δ*_90_ (mm)	*δ_u_* (mm)	*δ_u_/δ*_90_	*P*_max_/*P_y_*
(A) D21.7-L200-A15 (external diameter = 21.7 mm)	537.9	369.7	484.1	8.2	16.1	2.0	1.5
(B) D42.7-L200-A15 (Reference, external diameter = 42.7 mm)	775.5	482.0	698.0	9.5	27.2	2.8	1.6
Ratio (A/B)	0.69	0.77	-	-	-	0.70	0.94

**Table 9:** Results of push-out test (D42.7-L150-A15).

Specimens	No.	*P*_max_ (kN)	*P_y_* (kN)	*P_RK_* (kN)	*δ*_90_ (mm)	*δ_u_* (mm)	*δ_u_/δ*_90_	*P*_max_/*P_y_*
D42.7-L150-A15	1	750.2	535.4	675.2	9.6	21.9	2.3	1.4
	2	754.8	395.1	679.3	11.2	26.4	2.4	1.9
	3	718.6	462.3	646.7	9.5	25.2	2.6	1.6
	Avg.	741.2	464.3	667.1	10.1	24.5	2.4	1.6

**Table 10:** Comparison of characteristic values of different pipe length specimens (push-out test).

Specimens	*P*_max_ (kN)	*P_y_* (kN)	*P_RK_* (kN)	*δ*_90_ (mm)	*δ_u_* (mm)	*δ_u_/δ*_90_	*P*_max_/*P_y_*
(A) D42.7-L150-A15	741.2	464.3	667.1	10.1	24.5	2.4	1.6
(B) D42.7-L200-A15 (Reference)	775.5	482.0	698.0	9.5	27.2	2.8	1.6
Ratio (A/B)	0.96	0.96	-	-	-	0.86	1.00

**Table 11:** Results of pull-out test (D42.7-L200-A15).

Specimens	No.	*P* (kN)		*P*_pull_ (kN)		*δ*_90_ (mm)	*δ_u_* (mm)	*δ_u_/δ*_90_	*P*_max_/*P_y_*
		Max	Yielding	Max	Yielding				
D42.7-L200-A15 (Reference)	1	267.2	190.1	534.5	380.2	9.7	28.5	2.9	1.4
	2	260.5	166.0	521.0	332.0	12.6	36.4	2.9	1.6
	3	281.6	180.0	563.1	359.9	13.7	36.4	2.7	1.6
	Avg.	269.8	178.7	539.5	357.4	12.0	33.8	2.8	1.5

**Table 12:** Results of pull-out test (loop bar).

Specimens	No.	*P* (kN)		*P*_pull_ (kN)		*δ*_90_ (mm)	*δ_u_* (mm)	*δ_u_/δ*_90_	*P*_max_/*P_y_*
		Max	Yielding	Max	Yielding				
Loop bar-H16	1	304.7	231.6	609.4	463.2	7.1	12.2	1.7	1.3
	2	331.7	242.1	663.5	484.3	8.6	17.5	2.0	1.4
	3	327.8	240.7	655.5	481.4	8.0	14.7	1.8	1.4
	Avg.	321.4	238.1	642.8	476.3	7.9	14.8	1.9	1.3

**Table 13:** Comparison of characteristic values of different types of shear connector specimens (pull-out test).

Specimens	*P* (kN)		*P*_pull_ (kN)		*δ*_90_ (mm)	*δ_u_* (mm)	*δ_u_/δ*_90_	*P*_max_/*P_y_*
	Max	Yielding	Max	Yielding				
(A) Loop bar-H16	321.4	238.1	642.8	476.3	7.9	14.8	1.9	1.3
(B) D42.7-L200-A15 (Reference)	269.8	178.7	539.5	357.4	12.0	33.8	2.8	1.5
Ratio (A/B)	1.19	1.33	1.19	1.33	-	-	0.68	0.87

**Table 14:** Results of pull-out test (D42.7-L200-A10).

Specimens	No.	*P* (kN)		*P*_pull_ (kN)		*δ*_90_ (mm)	*δ_u_* (mm)	*δ_u_*/*δ*_90_	*P*_max_/*P_y_*
		Max	Yielding	Max	Yielding				
D42.7-L200-A10	1	251.6	178.1	503.1	356.2	9.7	28.5	2.9	1.4
	2	258.0	162.0	516.0	323.9	10.7	30.8	2.9	1.6
	3	258.7	154.7	517.4	309.4	13.8	36.5	2.6	1.7
	Avg.	256.1	164.9	512.2	329.8	11.4	31.9	2.8	1.6

**Table 15:** Comparison of characteristic values of different pipe insertion angle specimens (pull-out test).

Specimens	*P* (kN)		*P*_pull_ (kN)		*δ*_90_ (mm)	*δ_u_* (mm)	*δ_u_/δ*_90_	*P*_max_/*P_y_*
	Max	Yielding	Max	Yielding				
(A) D42.7-L200-A10 (angle = 10°)	256.1	164.9	512.2	329.8	11.4	31.9	2.8	1.6
(B) D42.7-L200-A15 (Reference, angle = 15°)	269.8	178.7	539.5	357.4	12.0	33.8	2.8	1.5
Ratio (A/B)	0.95	0.92	0.95	0.92	-	-	1.00	1.07

**Table 16:** Results of pull-out test (D42.7-L150-A15).

Specimens	No.	*P* (kN)		*P*_pull_ (kN)		*δ*_90_ (mm)	*δ_u_* (mm)	*δ_u_/δ*_90_	*P*_max_/*P_y_*
		Max	Yielding	Max	Yielding				
D42.7-L150-A15	1	169.6	102.2	339.2	204.4	6.9	19.4	2.8	1.7
	2	175.1	96.5	350.1	192.9	7.3	19.8	2.7	1.8
	3	156.5	126.2	312.9	252.4	4.2	15.3	3.7	1.2
	Avg.	167.0	108.3	334.1	216.6	6.1	18.2	3.1	1.5

**Table 17:** Comparison of characteristic value of different pipe length specimens (pull-out test).

Specimens	*P* (kN)		*P*_pull_ (kN)		*δ*_90_ (mm)	*δ_u_* (mm)	*δ_u_/δ*_90_	*P*_max_/*P_y_*
	Max	Yielding	Max	Yielding				
(A) D42.7-L150-A15	167.0	108.3	334.1	216.6	6.1	18.2	3.1	1.5
(B) D42.7-L200-A15 (Reference)	269.8	178.7	539.5	357.4	12.0	33.8	2.8	1.5
Ratio (A/B)	0.62	0.61	0.62	0.61	-	-	1.11	1.00

**Table 18:** Results of push-out test and numerical results.

Name	No.	*P*_max_ (kN)	*P_RK_* (kN)	*δ*_90_ (mm)	*δ_u_* (mm)	*δ_u_/δ*_90_
D42.7-L200-A15	1	773.3	696.0	9.8	28.0	2.9
	2	792.0	712.8	11.0	25.0	2.3
	3	761.2	685.1	8.2	28.5	3.5
	Avg.	775.5	698.0	9.6	27.2	2.8
D42.7-L200-A15 (FEM)	-	749.9	674.9	10.3	29.6	2.9

**Table 19:** Shear behavior characteristic of experimental and numerical results.

Name	*P*_max_ (kN)	*P_RK_* (kN)	*δ*_90_ (mm)	*δ_u_* (mm)	*δ_u_/δ*_90_
D42.7-L200-A15 (Reference)	749.9	674.9	10.2	29.5	2.9
D60.5-L200-A15 (T2.3)	837.8	754.0	10.5	24.1	2.3
D60.5-L200-A15 (T3.2)	901.2	811.1	7.8	23.9	3.1
D89.1-L200-A15 (T2.8)	1034.7	931.3	7.8	24.5	3.1
D89.1-L200-A15 (T3.2)	1186.5	1067.9	8.2	26.1	3.2
Loop bar-H16 (Loop bar)	918.8	826.9	10.2	14.2	1.4

**Table 20:** Results of pull-out test and numerical results.

Name	No.	*P*_max_ (kN)	*P_RK_* (kN)	*δ*_90_ (mm)	*δ_u_* (mm)	*δ_u_/δ*_90_
D42.7-L200-A15 (Reference)	1	267.2	534.5	9.7	28.5	2.9
	2	260.5	521.0	12.6	36.4	2.9
	3	281.6	563.1	13.7	36.4	2.7
	Avg.	269.8	539.5	12.0	33.8	2.8
D42.7-L200-A15 (FEM)	-	256.4	512.8	8.5	39.2	4.6

**Table 21:** Pull-out behavior characteristic of experimental and numerical results.

Name	*P*_max_ (kN)	*P_RK_* (kN)	*δ*_90_ (mm)	*δ_u_* (mm)	*δ_u_/δ*_90_
D42.7-L200-A15 (Reference)	256.4	512.8	8.7	38.5	4.4
D60.5-L200-A15 (T2.3)	285.1	570.2	7.2	31.4	4.4
D60.5-L200-A15 (T3.2)	304.0	608.0	8.1	32.2	3.9
D89.1-L200-A15 (T2.8)	334.3	668.6	9.6	33.1	3.4
D89.1-L200-A15 (T3.2)	356.6	713.2	9.3	32.3	3.5
Loop-H16 (Loop bar)	321.4	642.8	7.9	14.8	1.9
